# Cellular and Molecular Neuro-Bone Cell Interactions Drive Alveolar Bone Remodeling During Orthodontic Mechanical Loading

**DOI:** 10.7150/ijbs.129449

**Published:** 2026-02-26

**Authors:** Xinyi Fang, Chi Liao, Jiamin Wan, Chunmiao Jiang, Yating Yi, Jun Wang, Qianming Chen, Jiu Lin, Xiaoyan Chen

**Affiliations:** 1 Stomatology Hospital, School of Stomatology, Zhejiang University School of Medicine, Zhejiang Provincial Clinical Research Center for Oral Diseases, Key Laboratory of Oral Biomedical Research of Zhejiang Province, Cancer Center of Zhejiang University, Engineering Research Center of Oral Biomaterials and Devices of Zhejiang Province, Hang Zhou, China.; 2 State Key Laboratory of Oral Diseases & National Center for Stomatology & National Clinical Research Center for Oral Diseases, West China Hospital of Stomatology, Sichuan University, Chengdu, China.; 3 Department of Orthodontics, the Affiliated Hospital of Qingdao University, Qingdao, China.

**Keywords:** Neuron-bone cell signaling, orthodontic tooth movement, alveolar bone remodeling, sensory and autonomic nerves

## Abstract

Orthodontic tooth movement (OTM) is a biomechanically driven process governed by dynamic cellular and molecular signaling interactions between neural and skeletal systems. This review synthesizes current evidence on neuron-bone cell crosstalk and the coordinated involvement of immune and vascular components in regulating alveolar bone remodeling during OTM. Key neural contributors include sensory neurons (nociceptors), autonomic neurons, central nervous system (CNS) circuits, and Schwann cells, which communicate with osteoblasts, osteoclasts, and periodontal ligament cells to modulate their proliferation, differentiation, and functional activity. These interactions are mediated by defined signaling pathways, including neuropeptide signaling (CGRP-CLR, SP-NK1, NGF-TrkA, BDNF-TrkB), axon guidance signaling (Sema3A-PlexinA/Nrp1), adrenergic signaling (β2-AR-dependent pathways), and intracellular cascades such as Rac1-β-catenin, RhoA/ROCK2, and Notch3. Sensory nerves function as primary initiators by releasing neuropeptides that promote osteoclastogenesis in pressure zones and osteogenesis in tension zones, while simultaneously shaping local immune responses and vascular remodeling. The autonomic nervous system exerts context-dependent regulation, with sympathetic signaling favoring bone resorption and parasympathetic pathways emerging as modulators of osteogenesis and neurovascular homeostasis. CNS circuits integrate sensory and autonomic inputs to coordinate OTM kinetics and pain perception. Together, these neuro-osteogenic signaling networks define mechanistic targets for improving orthodontic outcomes and pain management via neuromodulation.

## Introduction

Bone is a dynamic tissue that constantly adapts its mass and architecture in response to mechanical loading. The nervous system, including the central nervous system (CNS) and the peripheral nervous system (PNS) has been identified as a critical regulatory system for bone remodeling 1. The CNS consists of brain and spinal cord, whereas the PNS acts as a fundamental connection between the CNS and the skeleton, and is subdivided into the sensory nervous system and the autonomic nervous system (ANS) 2, 3. The peripheral ANS is further subdivided into the sympathetic nervous system (SNS) and the parasympathetic nervous system (PSNS), which exert distinct effects on the targets organs. Additionally, autonomic nerves are composed of preganglionic and postganglionic neurons 4. Sensory nerves modulate bone remodeling primarily via neurotransmitters like calcitonin gene-related peptide (CGRP) and substance P (SP), and they also contribute to neurovascular coupling in bone homeostasis. SP exerts dual effects: it promotes terminal differentiation of osteoblasts, yet activates the nuclear factor κB (NF-κB) pathway in osteoclast precursors to promote bone resorption. Additionally, CGRP inhibits osteoclast differentiation by suppressing RANKL activation. Beyond sensory inputs, the SNS exerts a predominantly negative regulatory effect on the skeletal system by releasing norepinephrine to activate α- and β-adrenergic receptors, specifically promoting bone resorption and inhibiting bone formation. In contrast, the PSNS modulates the skeletal system in a positive regulatory manner, it releases acetylcholine to activate acetylcholine receptors (AChR), thereby enhancing bone formation and suppressing bone resorption. Collectively, these peripheral neural pathways are coordinated by the CNS, which serves as a central integration hub that processes systemic and peripheral sensory signals related to bone homeostasis, and modulates downstream autonomic and sensory neural outputs to coordinate bone formation and resorption throughout the skeleton 5.

Besides the direct interaction of nervous system and bone, the nerve-immune axis and neovascularization form another two core regulatory networks that orchestrate bone homeostasis. The nerve-immune axis mediates bidirectional crosstalk between neural cells and immune cells, neuropeptides and neurotransmitters regulate osteoblast and osteoclast functions through modulation of immune cells and cytokine secretion 6. Meanwhile, neurovascularization promote angiogenesis and vascular permeability via neurogenically derived factors, providing nutritional support and a functional microenvironment for bone remodeling processes 7.

These general neural regulatory principles provide a foundational framework for interpreting alveolar bone remodeling during orthodontic tooth movement (OTM). Alveolar bone is a unique tissue characterized by dense nerve distribution within its trabecular bone, periosteum, tooth apex, and the periodontium. This region is predominantly innervated by sensory nerves originating from the trigeminal ganglion (TG) and by a minor contribution from the autonomic nerves. OTM is a mechanical force-driven adaptive response of alveolar bone: sustained controlled forces create distinct pressure and tension zones in the periodontal ligament (PDL) and adjacent alveolar bone, while concurrently inducing treatment-associated pain 8. Mechanotransduction of these mechanical stresses drives bone apposition on the tension side and resorption on the compression side, thereby facilitating socket remodeling to accommodate tooth movement 9, 10. During OTM, mechanical strain on periodontal tissues distorts nerve fibers, triggering neurotransmission and local neurotrophins release 11. Notably, neurotransmitter and neuropeptide signaling occurs primarily via nonsynaptic mechanisms and intercellular junctions, and these bioactive molecules critically mediate key physiological processes in OTM, including pain transduction, inflammatory responses, and dynamic remodeling of the PDL and alveolar bone 12-14.

**Figure 1:**
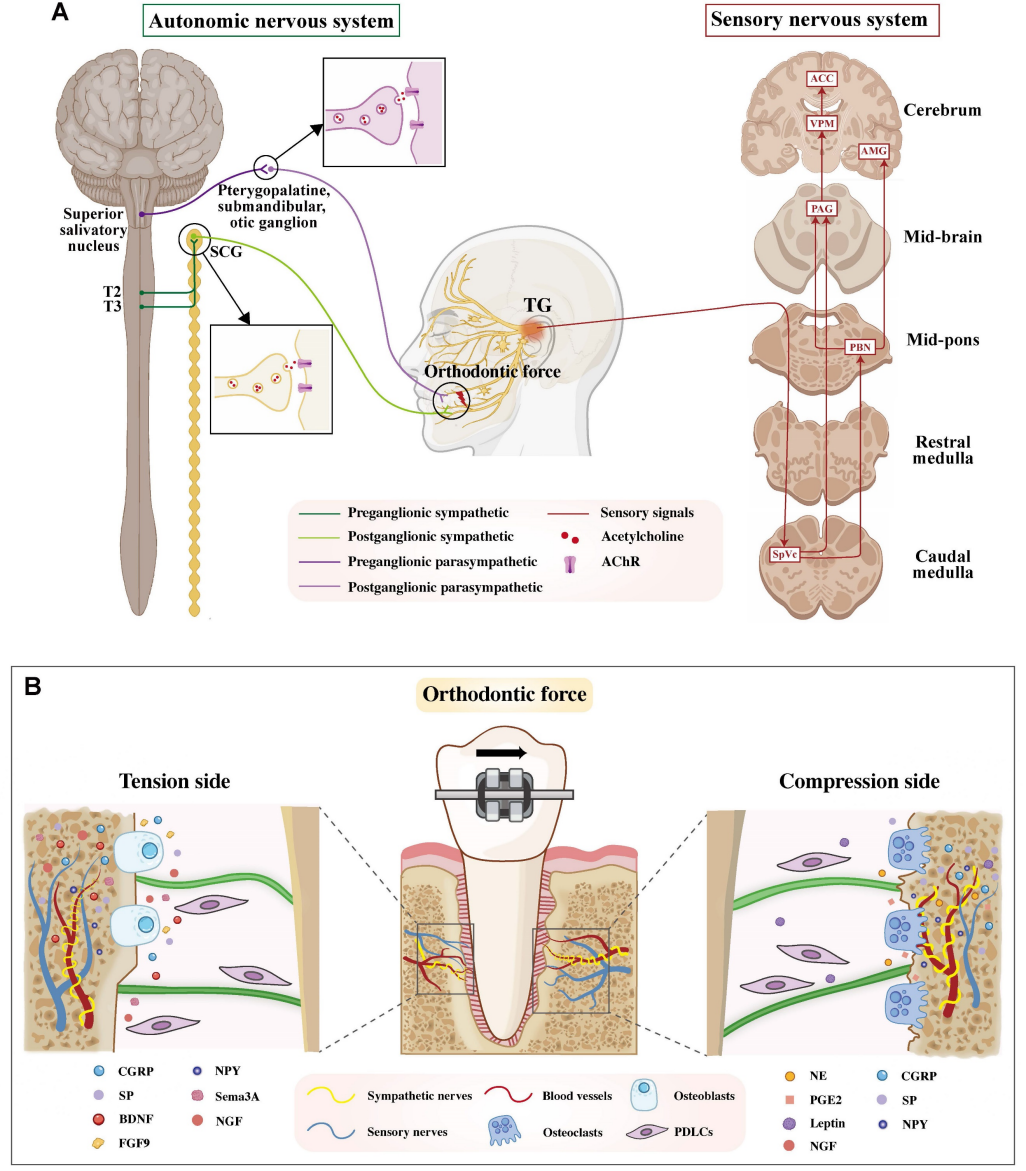
** A.** Schematic diagram of peripheral and central nervous system involved in OTM. The sympathetic and parasympathetic branches (synapsing in the SCG and pterygopalatine/submandibular/otic ganglia, respectively) regulate physiological functions during OTM. Sensory afferents from the TG transmit orthodontic force signals to the spinal trigeminal nucleus caudalis (SpVc), which then relays information to the thalamus and limbic system for pain perception. **B.** Schematic of periodontal innervation and neurotrophic factor distribution on compression and tension sides during OTM. The periodontal tissue is innervated by sensory nerves and perivascular sympathetic fibers. Orthodontic force-induced remodeling on the tension and compression sides is regulated by distinct neurotrophic factors. Abbreviations: SCG, superior cervical ganglion; AChR, acetylcholine Receptor; TG, trigeminal ganglion; ACC, anterior cingulate cortex; AMG, amygdala; PAG, periaqueductal gray; SpVc, spinal trigeminal nucleus caudalis; PBN, parabrachial nucleus; VPM, ventral posteromedial; NE, neurotransmitter norepinephrine; SP, substance P; CGRP, calcitonin gene-related peptide; Sema3A, Semaphorin 3A; NGF, Nerve Growth Factor; VEGF, vascular endothelial growth factor; BDNF, Brain-derived neurotrophic factor; PGE2, Prostaglandin E2; NPY, Neuropeptide Y; VIP, Vasoactive intestinal peptide; FGF, Fibroblast growth factor.

The nervous system orchestrates bone remodeling during OTM by targeting three basic functional cell types, osteoblasts, osteoclasts and periodontal ligament cells (PDLCs). This regulatory effect is mediated by the secretion of a diverse array of neural factors, including neurotransmitters, neuropeptides, neurotrophins, and neuronal guidance factors, alongside the involvement of nerve-resident cell components 8, 12. The coordinated behavior of these cell types ultimately governs the dynamic remodeling of alveolar bone throughout the OTM process. In this review, we have summarized and highlighted the latest advances in elucidating the critical roles of the nervous system in regulating periodontal tissue homeostasis and alveolar bone remodeling during OTM (Table 1). This work aims to provide novel insights into promising therapeutic targets for enhancing OTM efficiency and alleviating treatment-related pain, thus offering a theoretical basis to guide future basic and clinical research in this field (Figure 1).

## Peripheral and central nervous system involving in OTM

The CNS analyzes and integrates sensory inputs to generate coordinated responses to orthodontic force. During OTM, the PNS plays a pivotal role in transmitting periodontal sensory signals to the brain via the sensory nerve system, and in conveying regulatory commands from the brain to the periodontal tissues via the ANS during OTM.

Sensory nerves are densely distributed throughout the periodontal tissues, including the periosteum and alveolar bone compartments, where they transduce diverse noxious mechanical, thermal, and chemical stimuli. Among these, orofacial nociceptive neurons, a specialized subclass of primary sensory neurons residing in the TG and its branches, are uniquely responsible for detecting such harmful stimuli originating from various orofacial structures, including the periodontium. Approximately one-third of the periodontal afferents are reported to be nociceptive neurons 11. Orthodontic force application elicits a sterile inflammatory response in the PDL and alveolar bone, which activates local nociceptors. The generated afferent pain signals are first transmitted by the cell bodies of primary sensory neurons residing in the TG. Their central axons then project to and synapse within the caudal subnucleus of the spinal trigeminal nucleus (SpVc), a pivotal brainstem region dedicated to processing craniofacial nociception. From this key relay hub, pain signals are relayed rostrally via two principal pathways: the trigeminothalamic tract to the ventral posteromedial (VPM) nucleus of the thalamus for sensory-discriminative processing, and parallel projections via the parabrachial nucleus to limbic structures (e.g., the amygdala and anterior cingulate cortex) for affective-motivational processing 4, 15, 16. The integrated pathway activation ultimately culminates in the perception of spontaneous pain and mechanical allodynia during mastication, a prevalent and undesirable side effect of clinical orthodontic treatments 17, 18 (Figure 1).

In addition, the ANS also exerts a crucial role in the neural regulation of OTM. Sympathetic innervation of the cephalic region arises from preganglionic neurons in the intermediolateral horn of the upper thoracic spinal cord (T2-T3). Their cholinergic axons, employing acetylcholine (ACh) as the primary neurotransmitter, exit via the anterior roots, ascend the sympathetic chain, and synapse with postganglionic neurons within the superior cervical ganglion (SCG) 19. Following synaptic transmission, the postganglionic fibers from the SCG project to and innervate the periodontal tissues, where their principal role is mediated by the release of norepinephrine (NE) 20. In contrast, parasympathetic fibers originating from the pontine superior salivatory nucleus synapse in the pterygopalatine, submandibular, or otic ganglion, and their postganglionic fibers subsequently connect with the maxillary and mandibular branches of the trigeminal nerve to innervate the periodontium 21 (Figure 1).

## Sensory nerves and bone remodeling of OTM

Beyond their well-characterized role in pain perception, sensory nerves are also proven to exert a pivotal regulatory role in bone remodeling. For instance, TrkA Avil^-/-^ mice with congenital sensory denervation exhibited markedly reduced bone mass and a significant decrease in osteoblast numbers 22. Further research has elucidated the functional impact of modulating nociceptive nerves on alveolar bone remodeling. Non-invasive chemogenetic functional silencing of transient receptor potential vanilloid 1 (TRPV1)-expressing trigeminal afferents not only reduced activation of the TG but also attenuated the progression of bone loss in periodontitis 23. The tetrodotoxin-resistant (TTX-R) voltage-gated sodium channel (Nav1.8) serves as another hallmark expressed on nociceptors, making it a suitable molecular marker for manipulating a broad spectrum of these neurons. Genetic ablation of Nav1.8-expressing nociceptors in Nav1.8^Cre^/Diphtheria toxin A (DTA)^Lox^ mice resulted in a greater number of osteoclast and osteoblast precursors and an increased nuclear factor NF-kB ligand activator (RANKL)/osteoprotegerin (OPG) ratio in response to apical periodontitis, thereby accelerating bone resorption 24. Besides, it is well-established that the transection of inferior alveolar nerve (IAN), which is a major branch of the mandibular division of the trigeminal nerve and mainly primarily mediates sensation in the mandibular alveolar bone, leads to denervation of sensory nerves and subsequent mandibular destruction 25.

Sensory signals in OTM modulation have recently garnered considerable attention, given that periodontal tissues are densely innervated by TRPV1-expressing nociceptors which could secrete various neuropeptides in response to mechanical force, notably substance P (SP) and Calcitonin gene-related peptide** (**CGRP) 26. Studies in rats have explored the effects of IAN transection on neuropeptide expression and bone remodeling-related factors during OTM. Yamashiro *et al.* demonstrated that IAN transection depleted the immunoreactive (IR) to anti-CGRP and significantly reduced the osteoclast number and osteoclast surface in OTM 27. Yu *et al.* further illustrated that IAN-transection significantly reduced the expression levels of both CGRP and SP, as well as the OPG/RANKL ratio, during periodontal alveolar bone regeneration 28. Collectively, these findings indicate that sensory innervation influences both neuropeptide expression and the OPG/RANKL ratio, thereby governing periodontal alveolar bone regeneration processes. Intriguingly, while maxillary nerve transection significantly slowed OTM rate in the late phase of treatment, it did not induce a statistically significant reduction in the total amount of tooth movement achieved 29. The inconsistency in these conclusions may be attributed to differences in the specific sensory nerve branches transected and the magnitude of orthodontic force applied.

**Figure 2:**
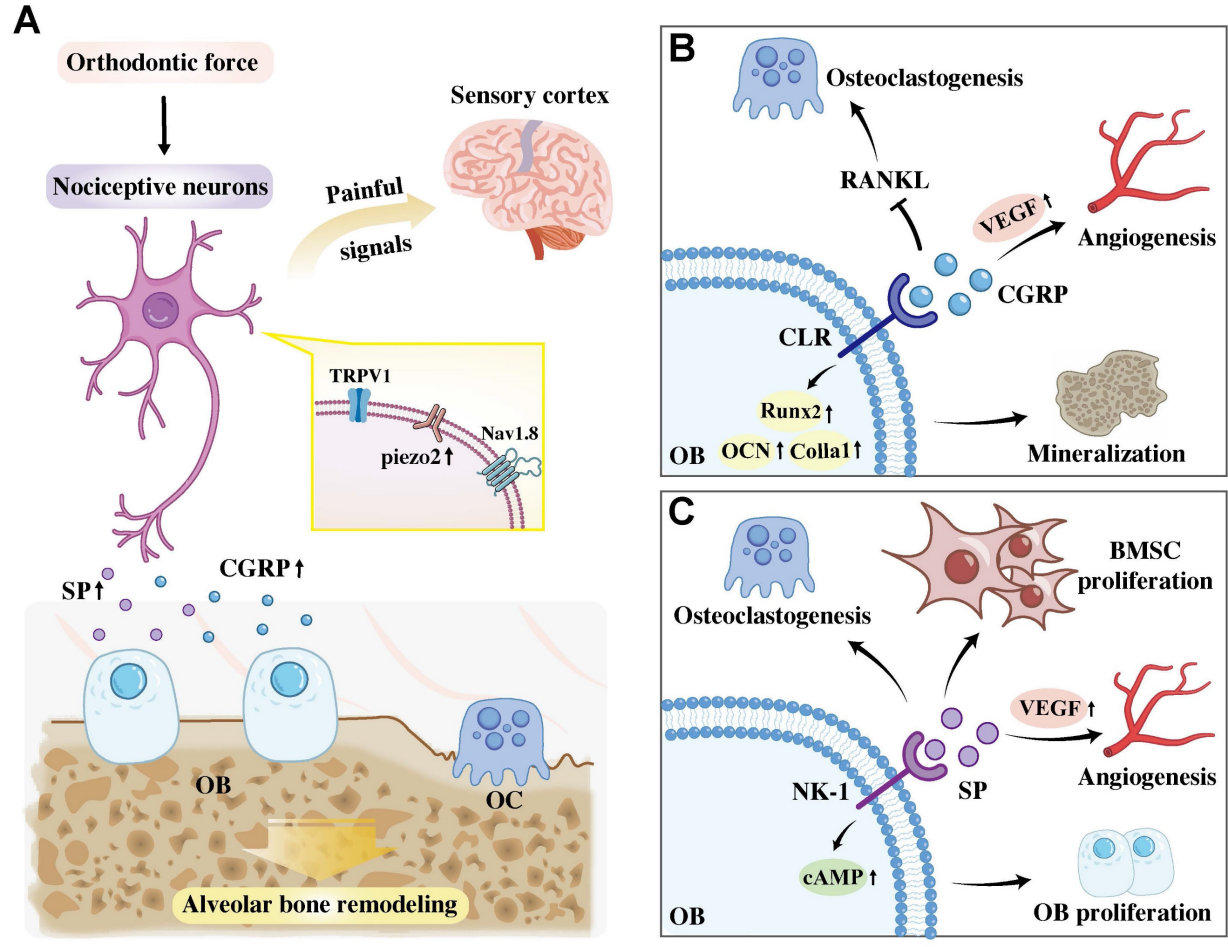
**A.** A dual-role mechanism of periodontal nociceptors in response to orthodontic force. Mechanical stimulation activates nociceptors, which not only transmit pain signals centrally but also activates Piezo2 channel and peripherally release SP and CGRP to directly regulate alveolar bone remodeling. **B.** CGRP binding to the CLR receptor on osteoblasts inhibits osteoclastogenesis by suppressing RANKL, promotes angiogenesis by stimulating VEGF production and promotes osteoblast mineralization by upregulating osteogenic gene expression. **C** SP acts via the osteoblastic NK-1 receptor to promote osteoclastogenesis, stimulate BMSC and osteoblast proliferation, and synergize with CGRP to drive angiogenesis. Abbreviation: TRPV1, transient receptor potential vanilloid 1; Nav1.8, tetrodotoxin-resistant voltage-gated sodium channel; OB, osteoblast; OC, osteoclast; VEGF, Vascular Endothelial Growth Factor; RANKL, nuclear factor NF-kB ligand activator; BMSC, bone marrow mesenchymal stem cell.

Mechanistically, Wang *et al.* elucidated that piezo ion channels, which are highly expressed in sensory neurons, mediate mechanosensation and mechanical pain perception during OTM. Their results reported that nociceptive neurons facilitate orthodontic force-induced alveolar bone remodeling via Piezo2 activation. OTM mechanical force activates Piezo2 via membrane stretching-induced conformational changes and synergistic regulation by cytoskeletal tension and phospholipid microenvironment. Specifically, conditional knockout of Piezo2 in TRPV1-lineage afferents significantly reduced OTM and decreased the number of osteoclasts 11. Thus, targeting these newly identified mechanisms involving nociceptive neurons represents a promising therapeutic strategy for modulating alveolar bone remodeling during OTM **(Figure 2A)**.

### CGRP

CGRP is the most abundantly secreted protein from sensory nerves, and its cognate receptor, the calcitonin-like receptor (CLR), is expressed on the surface of osteoblasts. Research has confirmed that CGRP upregulates osteogenesis-related genes (including *Runx2*, *Ocn* and *Col1a1*) and promotes the mineralization of osteogenic cells 30.

OTM upregulates the peripheral expression of the sensory neuropeptide CGRP 27, 31-36. It has been revealed that CGRP exerts a dual regulatory role during OTM: it inhibits osteoclastogenesis by suppressing RANKL activation and osteoclast differentiation, and it acts synergistically with SP to promote angiogenesis by upregulating vascular endothelial growth factor (VEGF) 37, 38. Consequently, CGRP induces vasodilation, stimulates new vessel formation, and enhances bone formation, which are all crucial for regulating blood flow and facilitating successful osteogenesis during alveolar bone remodeling 39 **(Figure 2B)**.

### Substance P

Substance P (SP) is a neuropeptide that is widely distributed throughout the nervous system, with its corresponding receptor, NK-1, found on the surface of osteoblasts 40. SP appears to play a dual role in alveolar bone remodeling. Some studies revealed that SP can stimulate osteoblast proliferation by promoting intracellular cAMP production 41. In the contrast, finding by Siddiqui *et al.* indicated that intragingival injection of SP induced osteoclast activation in alveolar bone. In line with this, the deletion of tachykinin precursor 1 (Tac1), a gene encoding SP, or treatment of the gingiva with SP antagonist, produced the opposite effects 42.

A coincident increase in SP and CGRP has been observed during OTM, with elevated levels of these neuropeptides spatially colocalized within the periodontal tissues 35, 43-45. Evidence indicates that sustained release of SP during tooth movement significantly accelerates tooth displacement by concurrently stimulating both osteoclast and osteoblast activity 8, 46, while acting synergistically with CGRP to promote angiogenesis via upregulating VEGF 38. Mechanistically, an *in vitro* study by An *et al.* demonstrated that SP enhances bone marrow mesenchymal stem cell (BMSC) proliferation and migration, a potential mechanism underlying its *in vivo* effects 46.

Furthermore, Symmank *et al.* uncovered a bidirectional SP-mediated crosstalk between PDLCs and sensory neurons, which potentially coordinates pain perception and bone remodeling during OTM 47. Specifically, mechanical compression induces PDLCs to secrete SP through upregulating Tac1, with secretion peaking at 24 hours. This fibroblast-derived SP then activates sensory neurons via the NK-1, characterized by increased neurite complexity, upregulated c-Fos expression, and calcium influx. Conversely, sensory neurons secrete SP that triggers PDLCs to produce pro-inflammatory cytokines (IL-1β, IL-6, TNF-α) and increase the RANKL/OPG ratio, thereby promoting osteoclastogenesis. Collectively, these findings suggest that SP and NK-1 are key targets for modulating OTM-related pain and optimizing alveolar bone remodeling **(Figure 2C).**

### Semaphorin 3A

The intricate relationship between the nervous and skeletal systems is also mediated by axon guidance molecules, including semaphorins and ephrins. Semaphorins primarily exert their biological functions through two groups of transmembrane receptors, namely plexin and neuropilins (Nrps) 48. Notably, Class III semaphorins, which depend on Nrps to form complexes with plexins, have been implicated in the bone remodeling process during OTM.

Semaphorin 3A (Sema3A), a member of the semaphorin family, is abundantly expressed in the VMH and functions as a crucial axon-guiding molecule that directs neuronal migration during CNS development 48. In skeletal tissue, Sema3A forms an osteoprotective complex with Plexin A by interacting with Nrp1^49^. This complex inhibits immunoreceptor tyrosine activation motif (ITAM) signaling and RhoA signaling pathways, thereby suppressing osteoclast differentiation induced by RANKL 50. Consequently, Sema3A inhibits bone resorption and promotes bone formation 51. Further evidence supporting its osteoprotective role from studies of Sema3A and Nrp-1 deficient mice, which displayed an osteoporotic phenotype characterized by reduced osteoblasts and impaired bone formation capacity 52.

**Figure 3:**
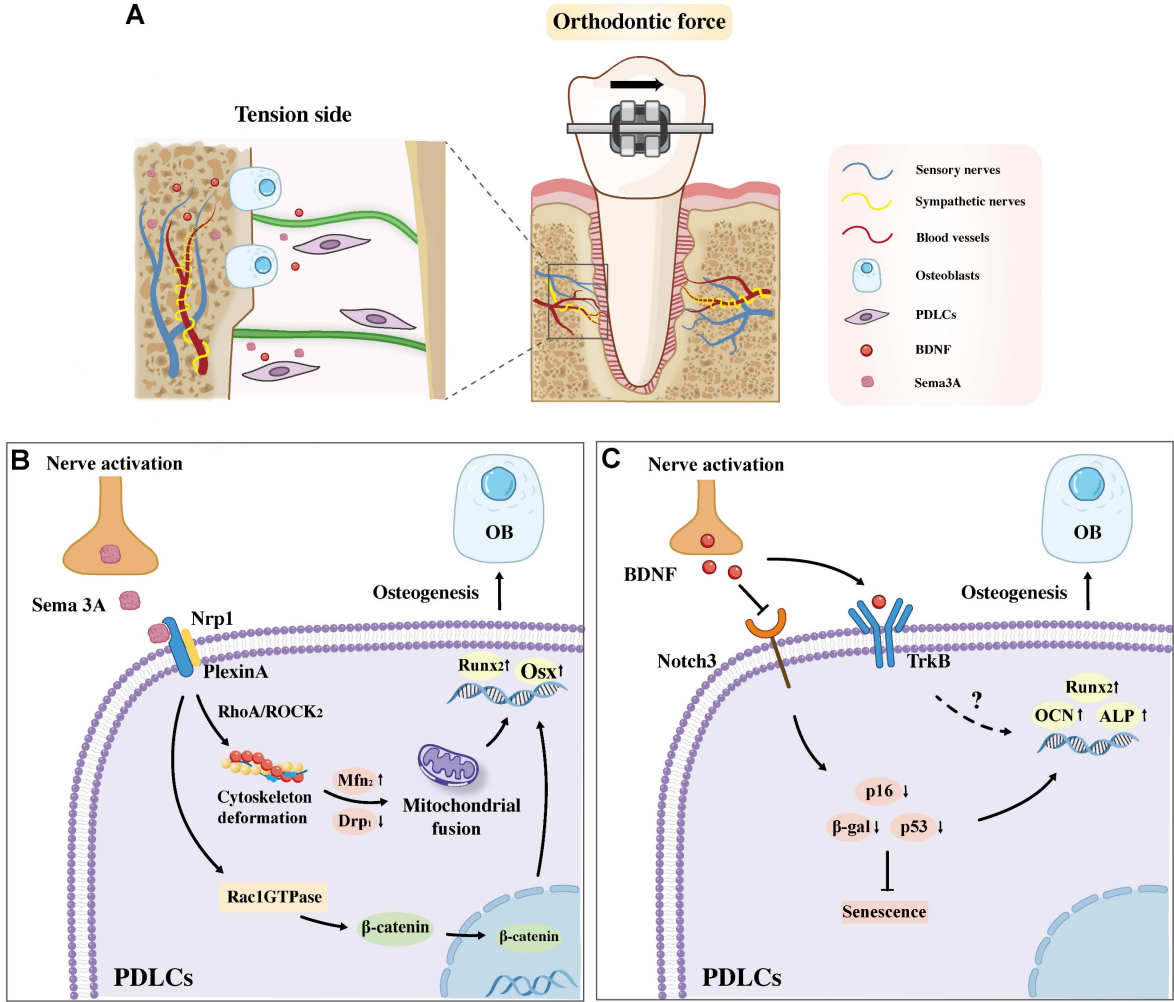
**A.** Sema3A and BDNF are implicated in regulating alveolar bone formation on the tension side under orthodontic force. **B.** Sema3A binding to Nrp1 on PDLCs promotes osteogenesis by activating Rac1-β-catenin-Wnt signaling and the RhoA/ROCK2 pathway for osteoblast genesis. **C.** BDNF promotes osteogenesis by inhibiting PDLC senescence via the Notch3 pathway and may also promotes osteogenesis through binding to the TrkB receptor. Abbreviation: PDLC, periodontal ligament cells; OB, osteoblast.

Given its dual capacity to promote osteoblast differentiation and suppress osteoclast activity, Sema3A has emerged as a crucial regulator of alveolar bone remodeling during OTM. Accumulating evidence highlights the pivotal role of Sema3A in driving alveolar bone formation on the tension side of OTM, primarily through its modulatory effects on PDLCs **(Figure 3A)**. For instance, Sen *et al.* reported that orthodontic force significantly upregulates the expression of Sema3A and its receptors, Nrp1 and Plexin A, in PDLCs on the tension side, while concurrently inhibiting their expression on the pression side. This tension side upregulation of the Sema3A-Nrp1/Plexin A axis enhances the osteogenic differentiation of human osteoblasts via activation of the Rac1GTPase and the nuclear translocation of β-catenin 53. Further mechanistic insights from Mei *et al.* demonstrated that mechanical loading activates the TG to promote sustained expression of Sema3A, which then interacts with PDLCs 54. This neuron-derived Sema3A upregulates Rho-associated protein kinase (ROCK2) protein in PDLCs, preventing excessive F-actin stretching under external force and maintaining mitochondrial dynamics through mitochondrial fusion, thereby facilitates the osteogenic differentiation of PDLCs **(Figure 3B).** Notably, exogenous Sema3A supplementation can reverse the inhibition of bone formation induced by orthodontic force and restore the osteogenic differentiation capacity of PDLCs.

The role of Sema3A in pain modulation has also been well documented. Sema3A exerts a neuropathic pain-alleviating effect by inhibiting the PI3K/Akt/mTOR signaling pathway, which subsequently reduces the phosphorylation level of eukaryotic translation initiation factor 2α (eIF2α) 55. Additionally, Sema3A plays a prominent role in relieving osteoarthritis-associated pain 56. Building on these findings, accumulating evidence has further identified a complementary modulatory role of Sema3A in OTM-induced nociception, as elucidated by Mei *et al.* 54. In the early stage of orthodontic force application, high expression of nerve growth factor (NGF) in the PDL activates sensory nerves and triggers acute pain. However, by day 3 post-force application, sustained upregulation of Sema3A inhibits the outgrowth of sensory nerves and induces their retraction in the PDL, leading to a reduction in neural excitability and the subsequent attenuation of OTM-induced pain. Sema3A functions as a multifunctional mediator that orchestrates mechanical load-induced bone formation and pain modulation during OTM, potentially serving as a promising target for optimized orthodontic therapies by simultaneously promoting optimal bone adaptation and alleviating treatment-associated discomfort.

### Nerve Growth Factor

Neurotrophins constitute a family of regulatory factors crucial for supporting various neural activities, including axonal growth, synaptic plasticity, cell survival, differentiation, and myelination. Beyond their well-established roles in neural system regulation, specific neurotrophins such as NGF and Brain-derived Neurotrophic Factor (BDNF) also mediate bone metabolism 1. Their regulatory functions in alveolar bone remodeling, particularly during OTM, have been extensively explored in recent studies.

As a key signal derived from sensory nerves, NGF coordinates bone development and adaptive remodeling by promoting reinnervation and regulating the migration of bone-related cells. NGF exerts its biological effects primarily through binding to high-affinity its receptors, tyrosine receptor kinase A (TrkA), which is abundantly expressed on the periosteal and endosteal surfaces of mature bone 57, 58. The NGF-TrkA signaling axis plays a pivotal role in directing skeletal innervation during development, as evidenced in the femur, where it regulates osteoprogenitor activity and vascularization. Conversely, disruption of NGF signaling has been shown to impair femoral innervation and reduce the secretion of VEGF 57.

Accumulating investigations have elucidated the crucial role of NGF in sensory signals transduction during OTM on both tension and compression sides. Long *et al.* discovered that exogenous NGF application in the periodontium can induce tooth mechanical hyperalgesia, while neutralizing antibodies against NGF effectively alleviate OTM-induced mechanical hypersensitivity 59. The work of Gao *et al.* further demonstrated that mechanical stimulation induces PDLC to express NGF, which is subsequently retrogradely transported to the TG 60. Within the TG, NGF specifically modulates tooth mechanical hyperalgesia by upregulating acid-sensing ion channel 3 (ASIC3), a mechanosensitive structure located in periodontal Ruffini endings. This finding is further supported by evidence that NGF knockdown led to reduced ASIC3 expression in the TG and alleviated hyperalgesia. However, the potential co-localization of the NGF receptor TrkA with ASIC3 remains to be demonstrated. Studies by O'Hara *et al.* and Long *et al.* have also revealed that OTM upregulates the expression of NGF and its receptors in periodontal tissues, triggering sprouting and infiltration of CGRP-positive nerve fibers 31, 59. Conversely, local administration of anti-NGF reduces tissue NGF levels and inhibits innervation of CGRP positive fibers. Given the established dual role of CGRP-positive sensory nerves in nociception and bone remodeling during OTM, NGF is postulated to indirectly regulate orthodontic bone adaptation and mechanical hyperalgesia by acting as a key inducer of CGRP positive nerve sprouting.

**Figure 4:**
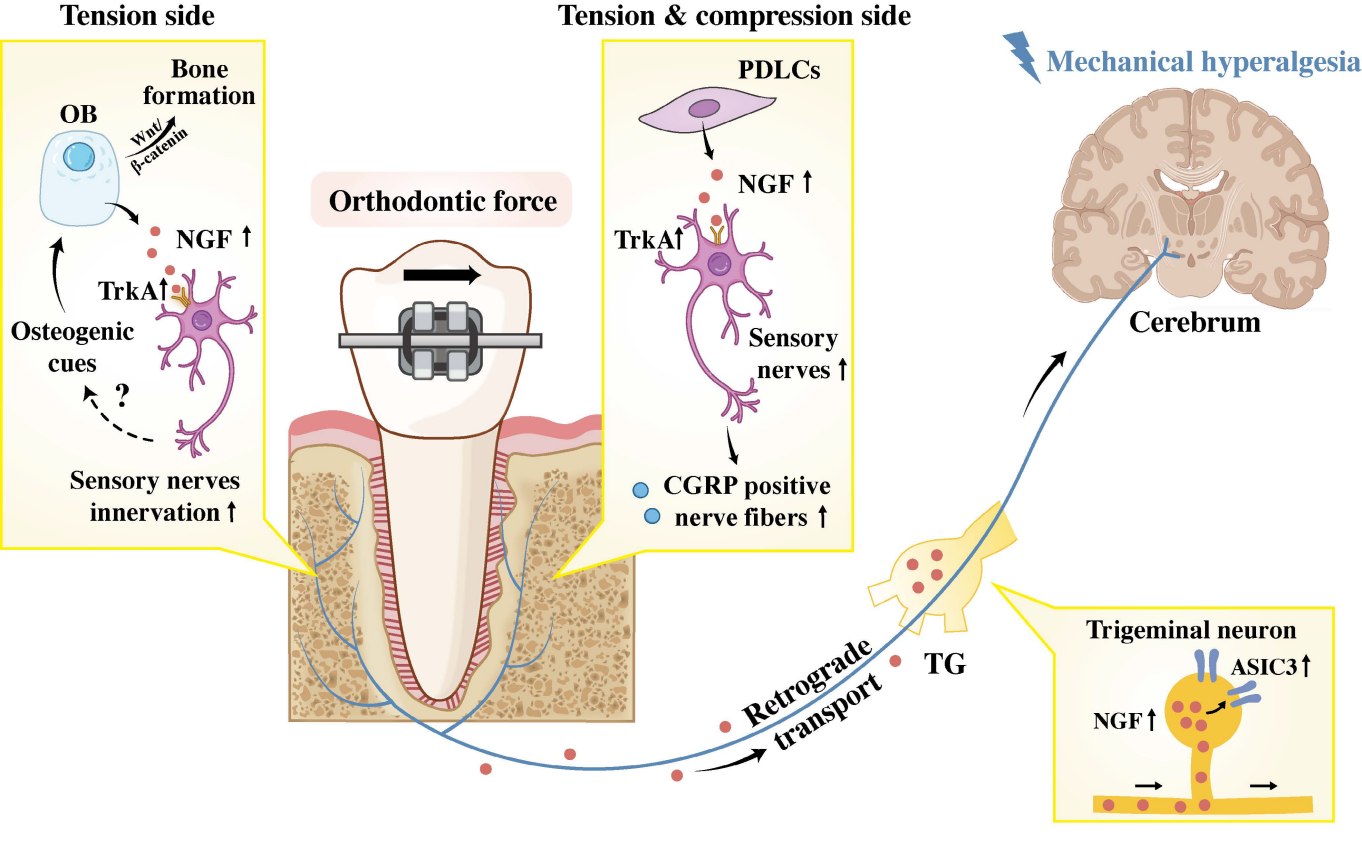
NGF coordinates bone remodeling and pain hypersensitivity during OTM. In the periphery, NGF (secreted by osteoblasts and PDLCs) binds TrkA on sensory neurons, promoting innervation and potentially enhancing bone remodeling via CGRP and osteogenic gene upregulation. Centrally, retrogradely transported NGF acts on trigeminal neurons, upregulating ASIC3 to facilitate central sensitization. Abbreviation: PDLC, periodontal ligament cells; OB, osteoblast; TG: trigeminal ganglion; ASIC3: acid-sensing ion channel 3.

Furthermore, the potential NGF-TrkA signaling pathway in sensory nerves for strain-adaptive bone remodeling has been explored. Tomlinson *et al.* demonstrated that mechanical stretching rapidly upregulates NGF expression in osteoblasts *in vivo*, which, in turn, activates TrkA in sensory nerves and triggers the subsequent release of osteogenic signals 61. These signals then enhance Wnt/β-catenin signaling in osteoblasts, thereby promoting bone formation **(Figure 4)**.

### BDNF

BDNF is widely expressed in the bone and periodontium, and exerts a prominent role in promoting skeletal cell differentiation via its specific receptor TrkB. This suggests that the BDNF-TrkB axis may play a potential regulatory role in bone formation and remodeling 62.

Notably, BDNF serves a dual function in orthodontic treatment, mediating both pain perception and periodontal tissue remodeling. On one hand, orthodontic force upregulates BDNF expression, evidenced in TG and PDLSCs 26. Furthermore, its salivary concentration in patients correlates with subjective pain intensity during early OTM 63. On the other hand, functional studies by Meng *et al.* revealed that BDNF is indispensable for the bone remodeling during OTM 64. Its knockdown accelerated the senescence of PDLCs and inhibited osteogenesis on the tension side specifically. The underlying mechanism involves BDNF suppressing the Notch3 pathway, which reduces cellular senescence (downregulation of β-gal, p16, p53) and subsequently enhances osteogenic capacity (upregulation of Runx2 and ALP), ultimately facilitating tooth movement **(Figure 3C)**. These findings position BDNF not only as a pain biomarker but also as a key anabolic regulator in OTM.

### Fibroblast growth factor

Fibroblast growth factor 9, a key member of the FGF family, and the FGF-FGFR signaling pathway play pivotal roles in neuro-osseous regulation 65. Skeleton-innervating somatosensory neurons (predominantly CGRP^+^ and Aβ-Field LTMR subtypes) secrete FGF9 as a core neurotrophic signal, which binds to FGFRs expressed on periosteal mesenchymal cells. This pathway mediates bidirectional crosstalk between nerves and bone: FGF9 not only upregulates dynamically in the reparative phase of skeletal injury, but also directly promotes mesenchymal cell proliferation and osteogenic differentiation, thereby facilitating bone repair 66.

Zhou *et al.* revealed that FGF9 functions as a critical negative regulator of bone remodeling by mediating crosstalk between osteocytes and preosteoblasts during OTM 67. As a paracrine factor predominantly secreted by osteocytes, FGF9 exerts its inhibitory effect through high-affinity binding to fibroblast growth factor receptor 2 (FGFR2) on preosteoblasts, triggering FGFR2 nuclear translocation to the nucleolus. This unique subcellular trafficking upregulates the osteogenic inhibitor ATF5 and downregulates the osteogenic promoter NR2F1, thereby suppressing preosteoblast differentiation and mineralization while promoting osteoclastogenesis. Notably, mechanical tension (e.g., facemask protraction) during OTM significantly downregulates FGF9 secretion from osteocytes, relieving its inhibitory constraint on osteogenesis and facilitating maxillary growth. Clinically, FGF9 overexpression is observed in patients with maxillary underdevelopment, and bone-targeted FGF9 overexpression in mice recapitulates this pathological phenotype, leading to shortened maxillary length and abnormal occlusion.

In summary, FGF9-FGFR2 signaling acts as a mechanosensitive switch in OTM, with mechanical tension counteracting FGF9's osteoinhibitory effects to enable adaptive bone remodeling, highlighting its potential as a therapeutic target for optimizing orthodontic outcomes in craniofacial skeletal abnormalities.

### Prostaglandin E2

Prostaglandin E2 (PGE2), derived from arachidonic acid, its biosynthesis depends on essential enzymes include cyclooxygenase (COX) and prostaglandin E2 synthase-1 (mPGES-1) 68. PGE2 has long been recognized as a potent intrinsic anabolic bone formation factor by binding to EP4 receptors on sensory nerves. It is secreted by osteoblasts in response to mechanical strain or changes in bone density. Activation of CREB signaling by the PGE2/EP4 ascending interoceptive activity in the ventromedial nucleus of the VMH downregulates sympathetic nerve activity as the descending interoceptive pathway, further influence bone formation 22.

Consistent with its regulatory role in bone remodeling, both animal and human studies have demonstrated that local administration of PGE2 significantly accelerated OTM by upregulating the RANKL/OPG ratio and osteoclastogenesis 69-71. However, the application of nonsteroidal anti-inflammatory drugs (NSAIDs), such as aspirin and ibuprofen, which are widely utilized for orthodontic pain management, may adversely affect OTM due to their ability to inhibit COX enzyme activity. *In vivo* studies consistently indicate that NSAIDs reduce the number of resorption lacunae and osteoclasts in the pressure area, consequently slowing the rate of OTM 72-74. These findings strongly suggest that disruption of the PGE2 signaling pathway can impair crucial bone resorption process. Besides, the NSAID indomethacin significantly exacerbated IL-1β-induced matrix metalloproteinases (MMPs) production by endothelial cells. Increased level of MMPs may cause aberrant remodeling of periodontal structures and affect normal OTM 75-77. In contrast, non-NSAID analgesics like acetaminophen, which do not or only minimally inhibit prostaglandin synthesis, do not exhibit these adverse effects and are therefore considered preferable for pain management during OTM.

**Figure 5:**
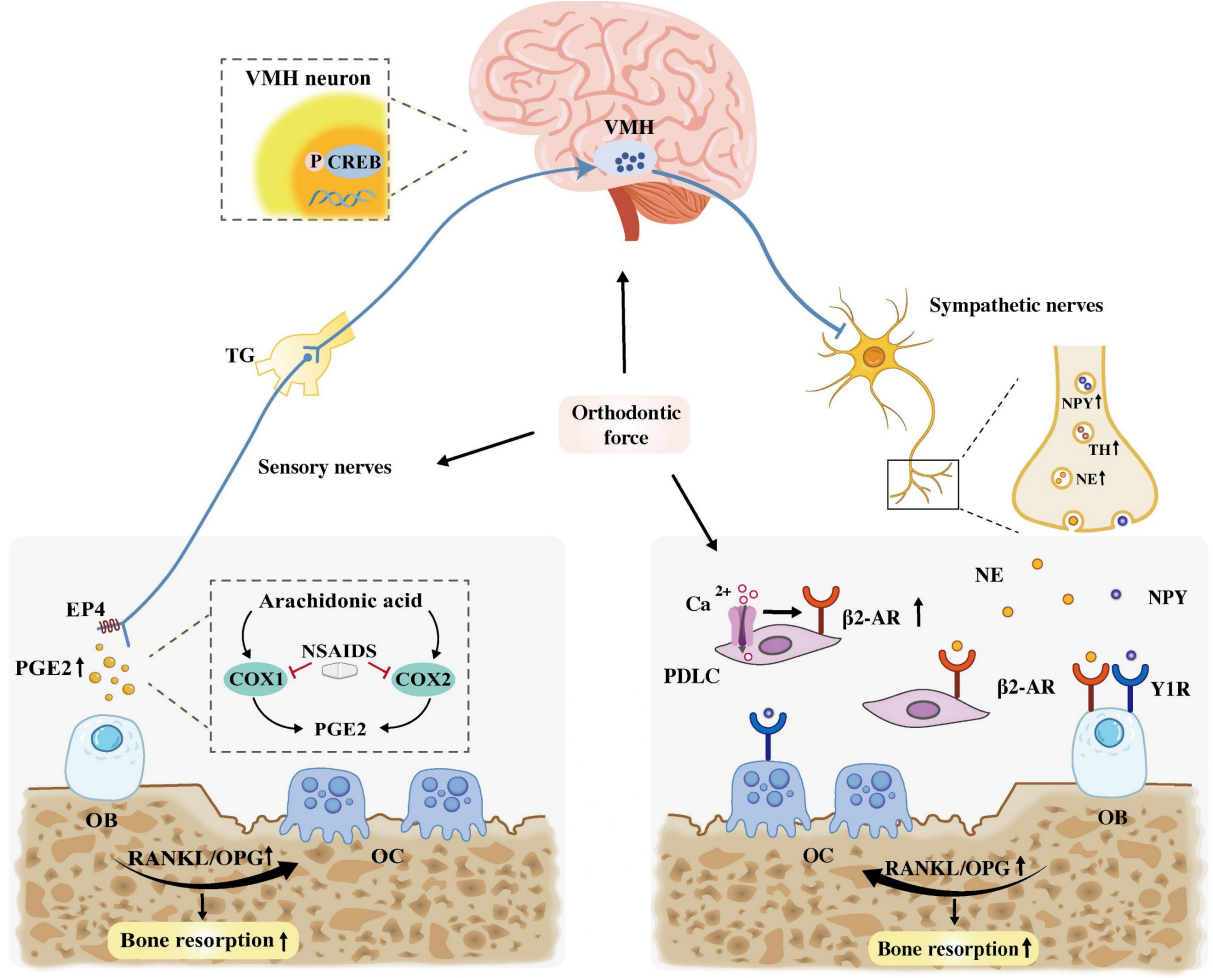
Sensory and sympathetic neural mechanisms regulating bone remodeling during OTM. On the one hand, PGE2 promotes bone resorption by elevating the RANKL/OPG ratio. Concurrently, it may also promote bone formation by binding to the EP4 receptor on sensory nerves, which activates the CREB pathway in the VMH to inhibit sympathetic tone. In contrast, NSAIDs attenuate OTM by inhibiting COX, the enzyme essential for PGE2 synthesis. On the other hand, orthodontic force stimulates the release of NE and NPY from sympathetic nerves, which activate β_2_-AR and Y1R on OBs/PDLCs. Additionally, force elevates intracellular Ca^2+^ in PDLCs, which directedly upregulating β_2_-AR expression in these cells. Collectively, sympathetic signals increase the RANKL/OPG ratio, leading to alveolar bone resorption. Abbreviation: VMH: ventromedial hypothalamus; OB, osteoblast; OC, osteoclast; NSAIDs, nonsteroidal anti-inflammatory drugs.

In summary, PGE2 probably exhibits a dual functionality in OTM. On one hand, it directly facilitates tooth displacement by promoting bone resorption in the pressure zone. On the other hand, it may indirectly enhance bone formation through central mechanisms that modulate sympathetic nerve activity, thereby improving the overall efficiency of OTM **(Figure 5)**.

## Autonomic nervous system and OTM

The ANS, encompassing its sympathetic and parasympathetic branches, exerts a dual influence on bone formation. Typically, sympathetic tone suppresses osteogenesis, whereas parasympathetic signaling appears to promote anabolic bone activity.

### SNS and bone remodeling of OTM

Sympathetic nerves are distributed throughout the periosteum, cortical bone, and bone marrow, where they maintain close anatomical association with blood vessels and typically wrapping spirally around the accompanying vascular structures 78. Within the bone microenvironment, sympathetic nerves express TH and secrete the neurotransmitter norepinephrine (NE). SNS has been shown to participate in the neural regulation of OTM. Specifically, dopaminergic stimulation of the VMH has been shown to increase the rate of OTM, whereas removal of the SCG reduces OTM efficiency 79. Furthermore, SNS signaling enhances osteoclastogenesis by modulation of the RANKL/OPG axis, thereby influencing OTM 80. Although neuropeptide Y (NPY) is co-expressed with sympathetic markers, its regulatory function in alveolar bone remodeling during OTM remains inconclusive.

#### Adrenaline and NE

Adrenaline is a potent neurotransmitter that exerts critical regulatory effect on bone cells. The SNS regulates bone metabolism primarily through tissue organ adrenergic receptors (ARs), particularly the β_2_-adrenergic receptor (β_2_-AR) 81. NE, the primary neurotransmitter released by sympathetic nerve terminals in bone, directly interacts with β_2_-AR expressed on the surface of various bone-associated cells, including osteoblasts, osteoclasts, and PDLCs. Although NE is the principal sympathetic neurotransmitter in bone, adrenaline also exerts a potent regulatory effect on bone cells via β_2_-AR. These adrenergic signaling interactions exert a significant negative regulatory effect on bone formation by shifting the bone remodeling balance toward resorption 82, 83.

Studies have demonstrated that mice deficient in β_2_-AR or dopamine β-hydroxylase (an enzyme required for NE synthesis) exhibit enhanced bone formation accompanied by reduced bone resorption 84. Furthermore, suppression of sympathetic signaling significantly reduces the rate of OTM in mice by inhibiting osteoclast activity. This inhibitory effect has been demonstrated through various interventions: pharmacological blockade using the β_2_-AR antagonist butoxamine or the non-selective β-blocker propranolol, and chemical sympathectomy induced by 6-hydroxydopamine (6-OHDA) 32, 85. Conversely, treatment with the adrenergic agonist isoprenaline accelerates OTM through enhanced osteoclast stimulation.

**Figure 6:**
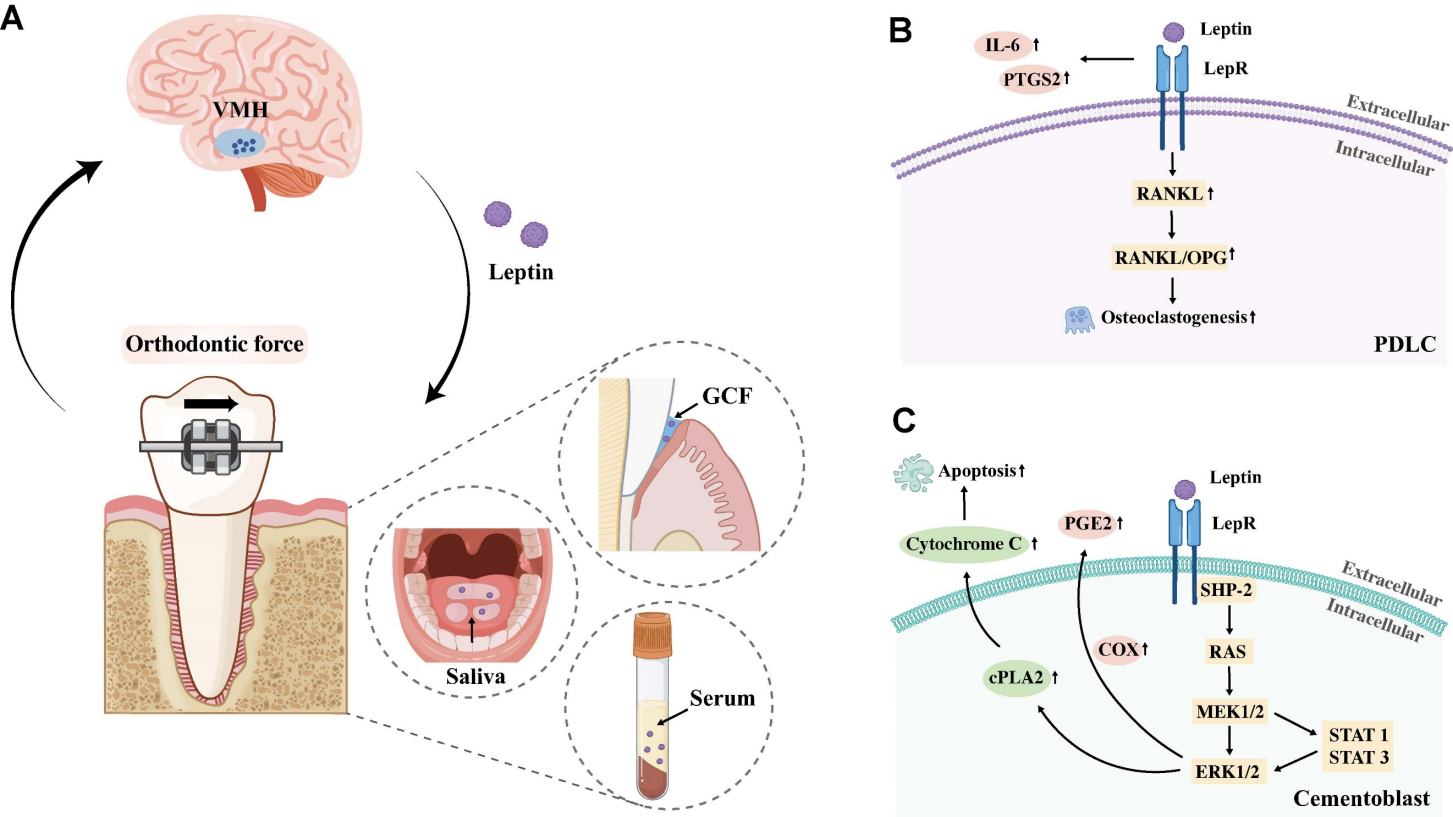
**A.** Schematic diagram showing altered leptin levels in gingival crevicular fluid, serum, and saliva during OTM, potentially associated with VMH regulation. Abbreviation: GCF, gingival crevicular fluid; VMH, ventromedial hypothalamus. **B.** Leptin/LepR signaling in PDLCs promotes inflammation and osteoclastogenesis. **C.** Leptin/LepR signaling in cementoblasts induces PGE2 release and apoptosis via ERK1/2.

Cao *et al.* further confirmed that the number of β_2_-AR-positive cells increased in the compressive side of the PDL following orthodontic force application in rats 79. Mechanistically, they elucidated that mechanical compressive force directly upregulated β_2_-AR expression in primary-cultured PDLCs via an elevation in intracellular Ca^2+^ concentration. Crucially, this upregulation of β_2_-AR in PDLCs subsequently increased the RANKL/OPG ratio, thereby promoting osteoclastogenesis, which, in turn, accelerated OTM through β_2_-AR-enhanced bone resorption **(Figure 6)**.

Collectively, these findings indicate that the β_2_-AR serve as a key regulator of OTM, acting as a convergence point for both sympathetic signaling and direct mechanical activation. By integrating these two regulatory inputs, β2-AR modulates osteoclast activity to facilitate tooth movement during OTM.

#### Neuropeptide Y

NPY is localized in sympathetic nerves throughout both the central and peripheral nervous systems 86. NPY plays a pivotal role in bone metabolism and remodeling, exerting its effects through direct and indirect signaling pathways mediated by Y1 and Y2 receptors, respectively 87. NPY primarily signals through Y1 receptors (Y1R), which are expressed on peripheral bone cells like osteoblasts and osteocytes. In contrast, the Y2 receptor (Y2R) is predominantly implicated in the central control of bone formation mediated by the VMH. Specifically, NPY-positive neurons in the arcuate nucleus (ARC) of the mouse VMH are coexpressed with leptin receptors, indicating a functional crosstalk between the NPY-Y2R pathway and leptin signaling in central bone regulation 88. *In vivo* evidence further confirmed this: Y2R and leptin double KO mice did not exhibit additional cancellous bone formation or volume increase 89; in contrast, Y2R deletion alone significantly doubled osteoblast activity in the presence of leptin 90.

While NPY is well recognized as a crucial negative regulator of bone formation through the VMH-dependent central pathway, its peripheral regulatory effects on bone metabolism remain controversial. NPY has been shown to exert bidirectional regulatory effects on the osteogenic potential of BMSCs by binding to Y1R. On one hand, it promotes bone resorption by upregulating the RANKL/OPG expression ratio 91. On the other hand, exogenous NPY treatment can enhance the expression of osteogenic genes (e.g., *Alp*, *Runx2*, and *Col1a1*) in BMSCs 92. Paradoxically, osteoblast activity and mineralization rate are significantly elevated in Y1R-deficient mice, which indicated the NPY-Y1R axis functions as a critical negative regulator of bone mass maintenance 93.

Investigations into NPY distribution during OTM have yielded contrasting observations. Kondo *et al.* reported an increase in sympathetic neuromarkers, including both TH and NPY, in the PDL during OTM 32. Conversely, Norevall *et al.* observed that while NPY-IR nerve fibers are present around blood vessels in the PDL, their distribution or density did not show significant changes in response to OTM 45 **(Figure 5).** Separately, NPY-containing nerve fibers originating from the TG have been documented to proliferate in the dental pulp and PDL following inferior alveolar nerve (IAN) injury in rats 94, 95. Despite these spatial and injury-induced dynamics, the precise functional contribution of NPY to the tissue remodeling processes in OTM remains largely elusive. A key direction for future investigation will be to determine the specific pathways through which NPY coordinates alveolar bone adaptation and pain modulation during OTM.

### PSNS and OTM

The parasympathetic nervous system (PSNS) is the other major branch of the ANS, which typically collaborates with SNS. As the mediator of the “rest-and-digest” response, the PSNS generally promotes bone anabolism and inhibits bone catabolism. However, its precise regulatory roles in OTM have not yet been clearly elucidated.

#### Vasoactive intestinal peptide

Vasoactive intestinal peptide (VIP), a key neurotransmitter of the PSNS, regulates bone formation, metabolism, and remodeling through its interactions with both osteoblasts and osteoclast 96. Previous studies have revealed complex effects of VIP: while it inhibits BMSC proliferation by binding to the VPAC1 receptor 97, it also promotes bone formation and regeneration by activating signaling pathways associated with osteogenesis 98. Despite these well-documented effects on bone cells, the specific role of VIP in OTM remains to be fully elucidated. This uncertainty is underscored by the absence of detectable changes in VIP-IR nerve fibers during OTM, as reported by Norevall *et al.* 45.

## Central nervous system and OTM

Kondo *et al.* demonstrated that OTM-activated sensory neurons enhance osteoclast activity and accelerate tooth movement via sympathetic nervous signaling 99. Specifically, their findings revealed that sensory nerve injury reduced CGRP and tyrosine hydroxylase (TH) immunoreactivity, concomitantly impairing osteoclast activity and attenuating overall tooth movement. Conversely, sympathectomy or ablation of the VMH diminished OTM without affecting CGRP immunoreactivity. The VMH is recognized as a key regulatory center within the CNS that controls bone metabolism, primarily through the SNS. Further evidence comes from functional magnetic resonance imaging (fMRI), a noninvasive and effective method for measuring blood oxygen level-dependent (BOLD) signals to visualize objective brain activity, has confirmed that interdental dehiscence induced by orthodontic appliances activated the VMH, alongside the sensorimotor and frontal association area in the human brain 100-103. Collectively, these results strongly highlight the CNS as a central integrator that mediates SNS-dependent regulation of orthodontic bone remodeling.

Taken together, current evidence robustly supports the existence of a sensory-CNS-sympathetic neural loop as a critical biological mechanism that directly modulates OTM.

### Leptin

Leptin, an adipocyte-derived cytokine binding to the leptin receptor (LepR), functions as both hormone and cytokine 104. In its hormonal capacity, it primarily acts as a hypothalamic modulator, regulating food intake, fat storage, and body weight maintenance 105. As a cytokine, however, leptin exhibits contrasting roles in bone remodeling: an indirect suppressive effect on bone formation mediated by the CNS via the VMH, and a direct stimulatory effect on osteoblasts peripherally 14.

Clinical evidence indicates that leptin levels in serum, saliva, and gingival crevicular fluid (GCF) correlate with tooth movement rates, and this association is further influenced by obesity status. However, research findings regarding changes in leptin concentration during OTM remain inconsistent. For instance, Dilsiz *et al.* and Srinivasan *et al.* collected gingival crevicular fluid (GCF) from orthodontic patients undergoing extraction and found that leptin concentration on the pressure side decreased significantly 7 days after orthodontic force application 106. In contrast, Soares Bonato *et al.* reported no significant differences in leptin concentration or tooth movement distance within the first week of force application 107. The impact of obesity on the association between leptin and OTM has been further explored. Elevated serum leptin levels in obese individuals have been shown to inhibit osteoclastogenesis via the RANKL/OPG pathway, thereby reducing OTM speed 108, 109. Consistently, Jayachandran *et al.* demonstrated that overweight patients exhibited higher salivary leptin levels accompanied by a decreased OTM rate 104. Nevertheless, conflicting evidence exists since some studies have reported a positive correlation between leptin levels and OTM speed in both normal-weight and overweight populations 104, 110. These contradictory findings are likely attributed to the distinct regulatory mechanisms of leptin from different sources. Serum leptin primarily exerts its effect through the VMH-sympathetic-osteogenic axis, indirectly inhibiting bone formation to modulate OTM 84. In contrast, locally derived leptin (from GCF and saliva) might act directly on periodontal cells via LepR, directly regulating osteoclastogenesis and osteoblast activity to influence alveolar bone remodeling.

The high-affinity LepR has been identified in PDLCs, cementoblasts and MSCs 111. Accumulating evidence demonstrates that leptin, by binding to LepR expressed on these cells, exerts multifaceted regulatory effects: it enhances PDLC-mediated inflammatory and osteoclastogenesis, promotes cementoblast activity to regulate inflammatory response, and potentially suppresses osteogenesis and promotes adipogenesis by altering MSCs differentiation via JAK2/STAT3 signaling 112. Collectively, these findings imply that leptin may serve as a pivotal regulatory factor in modulating OTM through its targeted actions on key periodontal and osseous cells. Regarding the specific mechanisms by which leptin regulates OTM, Schröder *et al.* demonstrated *in vitro* that leptin acts on PDLCs under mechanical strain. Their findings revealed that leptin amplifies the pro-inflammatory response, characterized by increased levels of IL-6 and PTGS2, and promotes osteoclastogenesis by significantly elevating the RANKL/OPG ratio 113. Additionally, Ruiz-Heiland *et al.* further reported that compressive stress induces leptin-mediated activation of the ERK1/2 pathway in cementoblasts, leading to cPLA2-driven PGE2 release and subsequent apoptosis, a process that may regulate the inflammatory response in the PDL during OTM 114. **(Figure 6)** However, the specific mechanism of leptin's action on OTM and the precise role of the nervous system in this process remain unverified by current studies, highlighting a key area for future investigation.

## Schwann cells

Schwann cells (SCs) are peripheral glial cells that are crucial for peripheral nerve regeneration 115, 116. Intriguingly, similar SCs reprogramming processes have also been observed in the context of alveolar bone regeneration. Zhang *et al.* demonstrated that SCs undergo dedifferentiation following adjacent alveolar bone injury and contribute to bone regeneration, primarily by accelerating the proliferation of alveolar skeletal stem cells (aSSCs). Specifically, SCs secrete factors that promote aSSCs proliferation via the PI3K-Akt and ERK/MAPK pathways 117.

Furthermore, Ito *et al.* reported that SCs can facilitate the regeneration and functional restoration of injured IANs 118. Trigeminal neuropathy, though reported as a rare complication, represents a challenging clinical issue secondary to OTM 119. Reported cases are primarily attributed to this neuropathy to orthodontic forces applied to premolar and molar teeth. This occurs due to the close proximity of the roots of these teeth to the inferior dental canal or mental nerve, leading to nerve damage from the application of force and torque. Such neuropathies highlight the critical need for effective nerve repair mechanisms, a process in which SCs play a pivotal role. Studies indicate that SCs reprogram to a repair phenotype following injury, becoming essential supporters of nerve repair. These repair SCs form a bridge-like structure post-peripheral nerve injury, guiding regenerating axons towards their targets 120.

In summary, Schwann cells hold considerable promise for promoting sensory nerve recovery and accelerating the healing of injured alveolar bone. However, their specific impact on the rate of OTM, particularly in modulating the balance between bone formation and resorption, requires further investigation.

## The role of the nerve-immune axis and neurovascularization in OTM

**Figure 7:**
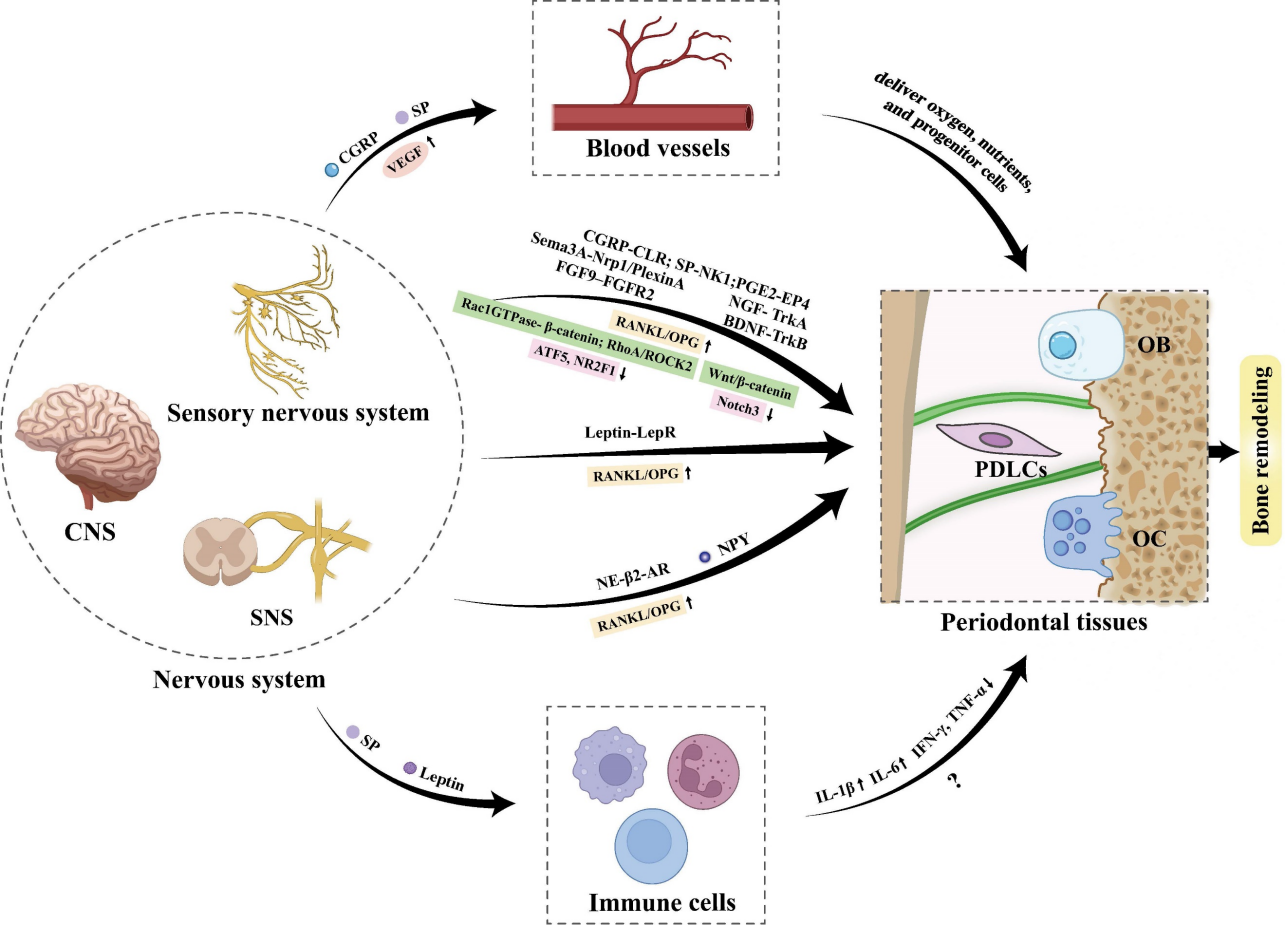
Crosstalk between nervous system, immune and vascular components and periodontal tissues during OTM.

The bidirectional crosstalk between the nervous system and immune cells is indispensable for efficient bone remodeling. The nerve-immune axis modulates bone regeneration primarily through two core modes of action: sensory and autonomic nerves shape the osteogenic immune microenvironment via central-peripheral signal conduction and local paracrine signaling, where key neuropeptides (e.g., CGRP, SP) and neurotrophins (e.g., NGF) orchestrate macrophage polarization, neutrophil recruitment, and MSCs immunomodulation, thereby fine-tuning the balance between inflammatory responses and bone remodeling processes 6 (**Figure 7**). In parallel, bone tissue homeostasis is equally dependent on the intricate bidirectional interplay between blood vessels and nerve fibers, a neurovascular crosstalk that underpins multiple physiological and pathological processes of the skeletal system. Blood vessels serve as the structural and functional foundation for bone and neural tissues by delivering oxygen, nutrients, and progenitor cells to sustain neural survival and the metabolic activity of bone cells; conversely, neural-derived factors (e.g., CGRP, VEGF, SP) secreted by sensory and autonomic nerves directly regulate vascular endothelial cell proliferation, migration, and angiogenesis, thus coupling neural signaling to vascular remodeling and ensuring the spatial and temporal coordination of bone tissue perfusion and remodeling 7.

In OTM, these regulatory mechanisms are activated in response to mechanical loading, leading to sterile inflammation and subsequent bone remodeling within PDL. On the neural-immune axis front, SP was also found to be elevated along with IL-1β in both compression and tension sites in the PDL of moving teeth 121, suggesting SP's potential role in modulating neuroinflammatory crosstalk to facilitate tooth displacement. Mechanistically, Lee *et al.* demonstrated that SP release from PDLCs induces C-C ligand 20 (CCL20) expression, and further revealed that SP regulates the macrophage inflammatory protein 3α/CCL20 ratio in PDLCs via heme oxygenase-1, thereby triggering CCL20-dependent inflammatory responses 122. Conversely, An *et al.* found that systemic administration of SP reduces the expressions of IFN-γ and TNF-α in periodontal tissues in the late phase of OTM, indicating a potential regulatory pattern of SP in OTM-associated inflammation 46. Beyond SP, leptin also modulates the immune response during OTM by promoting the expression of inflammatory mediators such as IL-6 113. Under hypoxic conditions, Gao *et al.* revealed that leptin inhibits reactive oxygen species (ROS)-mediated apoptosis of PDLCs via the ROS-hypoxia-inducible factor-1α (HIF-1α) pathway, while HIF-1α inhibition attenuates hypoxia-induced leptin upregulation and PDLC apoptosis 123.

Neurovascularization also plays an indispensable role in periodontal tissue adaptation during OTM. Notably, the elevated CGRP induced by mechanical loading acts synergistically with SP to promote angiogenesis by upregulating VEGF expression 37, 38. In animal models of OTM with IAN transection, the recovery of blood flow in the PDL and dental pulp is significantly delayed, accompanied by retarded recruitment of monocytes/macrophages, reduced osteoblastic activity, and a notable decrease in tooth movement efficiency 124-126. These findings further confirm that sensory nerves and their secreted neuropeptides play pivotal regulatory roles in modulating immune responses and neurovascular adaptation during OTM, but the explicit mechanisms require further exploration.

## Conclusion

OTM-associated alveolar bone remodeling is a complex process driven by biomechanical stimuli and coordinated via multi-dimensional molecular crosstalk among neural, skeletal, immune, and vascular components **(Figure 7)**. This review confirms that sensory neurons, autonomic neurons, CNS circuits, and SCs are core neural regulators, which target osteoblasts, osteoclasts, and PDLCs and interact with immune/vascular components to modulate OTM. Critical signaling pathways, including neuropeptide-, axon guidance molecule-, adrenergic-, and key intracellular cascades, underpin these regulatory interactions, orchestrating alveolar bone remodeling and the integration of pain perception.

**Figure 8:**
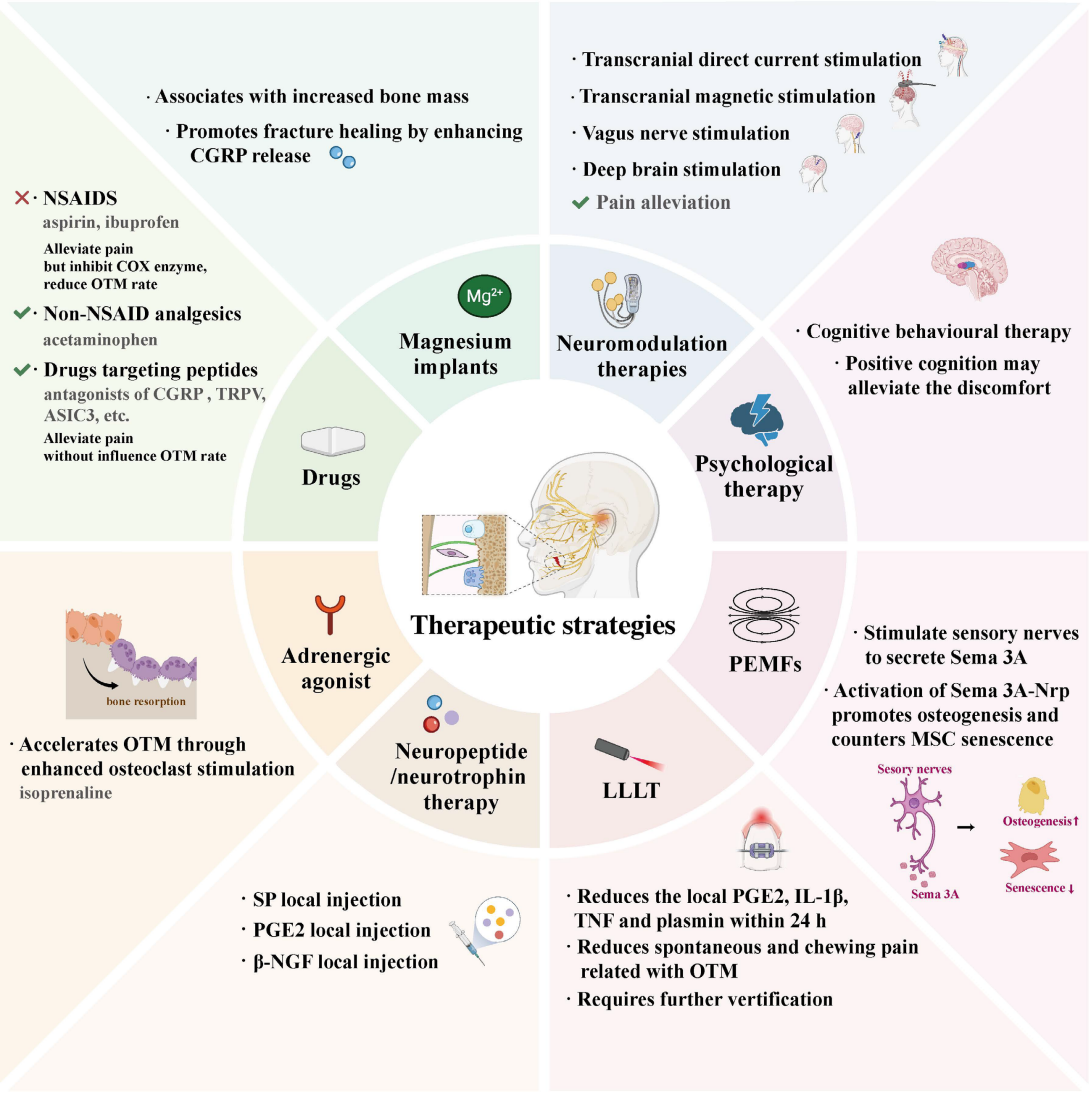
Innovative therapeutic strategies targeting neuro-bone signaling to optimize orthodontic tooth movement and reduce pain. LLLT, low-level laser therapy; PEMFs, pulsed electromagnetic fields.

Notably, these multi-dimensional neural interactions not only clarify the mechanistic basis of OTM regulation but also identify promising therapeutic targets, including key neuropeptides (CGRP, SP, NGF, BDNF), the β2-AR-dependent sympathetic axis, Sema3A-PlexinA/Nrp1 pathway, and SC plasticity **(Table 2)**. Targeting these molecules/pathways via precise neuromodulation holds great potential for enhancing OTM efficacy and alleviating treatment-related pain. For instance, drugs (specifically drugs targeting peptides), neuromodulation therapies (including transcranial magnetic stimulation, transcranial direct current stimulation, vagus nerve stimulation, etc.), psychological interventions, and low-level laser therapy (LLLT) are likely to be effective in alleviating orthodontic treatment-related pain 5, 127-129. On the other hand, adrenergic agonists, pulsed electromagnetic fields (PEMFs), neuropeptide-based therapies, and magnesium implants may facilitate the acceleration of alveolar bone remodeling during OTM 32, 46, 129-131 **(Figure 8)**.

However, targeting the nerve-bone axis for OTM optimization still faces some major translational challenges. First, most mechanistic evidence is from rodent and *in vitro* models, with insufficient well-powered human clinical trials validating clinical safety and efficacy. Second, patient heterogeneity in genetics, skeletal patterns and gingival biotypes leads to variable responsiveness to neuromodulatory interventions. Besides, chronic neuromodulation may disrupt physiological bone and neural homeostasis, posing long-term risks such as impaired bone remodeling and increased iatrogenic root resorption. These challenges underscore the necessity of conducting in-depth future investigations into the efficacy, safety, and underlying mechanisms of these novel interventions. Additionally, further studies are warranted to focus on the functional divergence of neuropeptides between the tension and compression zones, as well as the spatiotemporal switching of neural signaling cascades that regulate OTM.

## Figures and Tables

**Table 1:** Roles of neurogenic factors in orthodontic teeth movement (OTM).

Neurogenic factors		Source	Receptor	Cell lineages	Roles in OTM	References
Sensory nerve system	CGRP	Sensory nerves	CLR, RAMP1	Osteoblasts, osteocytes	Osteogenesis and angiogenesis	27, 31, 32, 34-36
	SP	Sensory nerves	NK-1	Osteoblasts, osteocytes	Osteogenesis and osteoclastogenesis	8, 46
	Sema 3A	Located on the growth cones and axons in dorsal root ganglia and cranial ganglia; Expressed by mesenchymal stem cells, osteoblasts, osteoclasts, and chondrocytes	Neuropilin,PlexinA	PDLCs, osteoblasts	Osteogenesis	48-54
	NGF	Schwann cells of peripheral nerves, endothelial cells, BMSCs and osteoblasts	TrkA, p75NTR	PDLCs, osteoblasts	Pain perception and osteogenesis	31, 57, 59-61
	BDNF	Schwann cells of peripheral nerves	TrkB	PDLCs, osteoblasts	Pain perception and osteogenesis	63, 64, 132
	FGF9	Sensory nerves, osteocytes	FGFR2	Osteoblasts	Inhibition of osteogenesis	67
	PGE2	Osteoblasts	EP4	Osteoblasts	Accelerate bone resorption and potentially promote bone formation	69, 72, 76, 133
SNS	NE	Adrenergic nerves	β2-AR	Osteoblasts, osteoclasts, PDLCs	Promote osteoclastogenesis and accelerate bone resorption	32, 79, 80, 82-85, 134
	NPY	Adrenergic nerves and osteocytes	Y1 & Y2	Osteocytes, osteoblasts	Increased during OTM, but the role is not clear	45, 94, 95, 135
PSNS	VIP	Cholinergic nerves	VPAC1	BMSCs	No significant changes have been found, and the role is not clear	45, 97, 98, 136
CNS	Leptin	Adipocytes	LepR	PDLCs, osteoblasts, cementoblasts	Osteoclastogenesis and inflammation	104, 106-110, 113, 114, 137
Resident cell components	Schwann cells	Originates from the neural crest cells	/	/	Proliferation of aSSCs and regeneration of injured IAN	117-120

**Table 2:** Possible therapeutic targets and specific molecular mechanisms mediating neuro-skeletal coupling in orthodontic bone remodeling.

Molecular and receptors	Pathway	Neuro and bone cell types involved	Effect on alveolar bone remodeling	Effect on pain	Level of evidence	Reference
TRPV1	/	Sensory neurons, osteoclasts	Inhibition and knockout of Piezo2 decreased OTM and the number of osteoclasts	Ablation and knockout of TRPV1 attenuated orthodontic pain	Animal (mice)	11, 17
Piezo2						
CGRP-CLR	RANKL/OPG	Sensory neurons, PDLCs, osteoblasts	Inhibits osteoclastogenesis, promotes angiogenesis	Pain interception	Animal (rats)	38
SP-NK1	RANKL/OPG	Sensory neurons, PDLCs, MSCs, osteoblasts	Accelerates OTM by upregulating osteoclastogenesis and mobilizing endogenous MSCs, promotes angiogenesis	Pain interception	Animal (rats) and *in vitro*	46, 47
Sema3A-Nrp1/PlexinA	Rac1GTPase- β-catenin	PDLCs, osteoblasts	Enhances the osteogenic differentiation of osteoblasts on the tension side	/	*In vitro*	53
	RhoA/ROCK2	PDLCs, osteoblasts	Facilitates the osteogenic differentiation of PDLCs on the tension side	Inhibits axon sprouting and attenuated orthodontic pain by day 3 post-force application	Animal (mice) and *in vitro*	54
NGF- TrkA	Wnt/β-catenin	Sensory neurons, osteoblasts	Promotes bone remodeling	Induce tooth mechanical hyperalgesia	Animal (mice)	60, 61
BDNF-TrkB	Alleviates the senescence of PDLSCs by inhibiting Notch3	PDLCs	Promotes osteogenesis on the tension side	Its salivary concentration in patients correlates with subjective pain intensity during early OTM	Human, animal (mice) and *in vitro*	63, 64
PGE2- EP4	RANKL/OPG	Sensory neurons, osteoblasts	Accelerates OTM by upregulating osteoclastogenesis	Pain interception	Human, animal	69-71
FGF9-FGFR2	Modulates the transcription factors ATF5 and NR2F1, which in turn downregulate FGFR2 protein expression and inhibits osteogenesis	Osteocyte, osteoblast	Tension signals reduce FGF9 secretion, thereby promoting osteogenic differentiation and facilitating maxillary development	/	Animal (mice) and *in vitro*	67
NE- β_2_-AR	RANKL/OPG	PDLCs	Accelerates OTM by upregulating osteoclastogenesis on the compression side	/	Animal (mice) and *in vitro*	1, 29, 32, 33
Leptin- LepR	RANKL/OPG	PDLCs	Promotes osteoclastogenesis on the compression side	/	*In vitro*	45, 46

## Abbreviations

ARs: adrenergic receptors
aSSCs: alveolar skeletal stem cells
BDNF: brain-derived neurotrophic factor
BMCs: bone marrow-derived cells
BMSC: bone marrow stromal cell
CGRP: calcitonin gene-related peptide
CLR: calcitonin-like receptor
CNS: central nervous system
COX: cyclooxygenase
fMRI: functional magnetic resonance imaging
FGF: fibroblast growth factor
FGFR: fibroblast growth factor receptor
GCF: gingival crevicular fluid
IAN: inferior alveolar nerve
IR: immunoreactive
LepR: leptin receptor
LLLT: low-level laser therapy
MMPs: matrix metalloproteinases
mPGES-1: prostaglandin E2 synthase-1
NE: neurotransmitter norepinephrine
NF-κB: nuclear factor κB
NGF: nerve growth factor
NPY: neuropeptide Y
Nrps: neuropilins
NSAIDs: nonsteroidal anti-inflammatory drugs
OB: osteoblast
OC: osteoclast
OPG: osteoprotegerin
OTM: orthodontic tooth movement
PDL: periodontal ligament
PDLCs: periodontal ligament cells
PEMFs: pulsed electromagnetic fields
PGE2: prostaglandin E2
PNS: peripheral nervous system
PSNS: parasympathetic nervous system
RANKL: nuclear factor NF-kB ligand activator
ROCK2: rho-associated protein kinase
SCG: superior cervical ganglion
SCs: Schwann cells
Sema3A: Semaphorin 3A
SNS: sympathetic nervous system
SP: substance P
SpVc: spinal trigeminal nucleus
Tac1: tachykinin precursor 1
TG: trigeminal ganglion
TH: tyrosine hydroxylase
TrkA: tyrosine receptor kinase A
TRPV1: transient receptor potential vanilloid 1
VEGF: vascular endothelial growth factor
VIP: vasoactive intestinal peptide
VPM: ventral posteromedial
Y1R: Y1 receptor
Y2R: Y2 receptor
β2-AR: β2-adrenergic receptor
